# Diffuse skin rash in tropical areas: Dengue fever or COVID-19?^☆^^☆☆^

**DOI:** 10.1016/j.abd.2020.10.001

**Published:** 2020-11-17

**Authors:** Isabelle Pastor Bandeira, Beatriz Sordi Chara, Giovani Meneguzzi de Carvalho, Marcus Vinícius Magno Gonçalves

**Affiliations:** a Department of Medicine, Universidade da Região de Joinville, Joinville, SC, Brazil; b Department of Medicine, Hospital Regional Hans Dieter Schmidt, Joinville, SC, Brazil; c Department of Neurology, Universidade da Região de Joinville, Joinville, SC, Brazil

**Keywords:** Betacoronavirus, Coronavirus infections, Dengue, Exanthema, COVID-19

## Abstract

There have been several clinical manifestations associated with SARS-CoV-2 infection since 2019, including dermatological signs and symptoms. In this article, the authors report a case of a previously healthy patient with COVID-19 who was mistakenly diagnosed with dengue fever due to a skin rash. By the time the patient's investigation was initiated, Joinville (Santa Catarina, Brazil) had approximately 5,000 confirmed cases of dengue fever and 1,700 cases of COVID-19 in 2020. Thus, the authors emphasize that in endemic regions such as Brazil, the two diseases must be considered until proven otherwise. Finally, the authors warn of the possibility of co-infection with these two viruses in regions that are facing both epidemics at the same time.

A 26-year-old male patient arrived at the emergency room (ER) complaining of fever, cough, myalgia, and mild dyspnea for seven days. The patient had no previous medical history and did not use any medication. Physical examination revealed a heart rate of 79 bpm, respiratory rate of 18 rpm, a systolic blood pressure of 137 mmHg, oxygen saturation of 99%, and a temperature of 96.8 °F. Considering the COVID-19 pandemic, the authors started the protocol for this infection: symptomatic treatment, a nasopharynx sample collection for SARS-CoV-2 RT-PCR, and social isolation for 14 days, counting from symptom onset. Two days later, the patient returned to the ER with a pruritic maculopapular confluent rash over the neck, chest, upper, and lower limbs (Fig. 1). At this moment, he was diagnosed with dengue fever (DF) – a viral infection endemic in tropical regions, which also manifests with flu-like symptoms, with the appearance of a diffuse skin rash between three to six days after the onset of fever.^1^ Nonetheless, the patient remained in social isolation until the test result for COVID-19 was made available.

**Figure 1:**
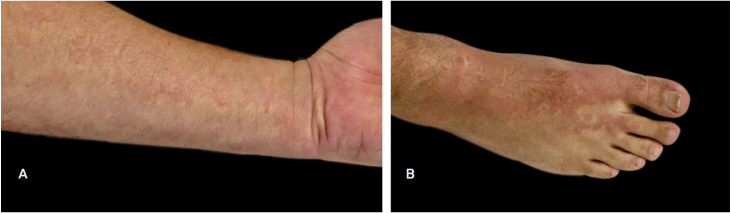
Confluent rash presented by the patient nine days after the onset of respiratory symptoms. The skin lesions persisted for three days, and the pruritus improved with the use of antihistamines. (A) Lesions on the forearm were the first to appear, with macular characteristics; (B) On the lower limbs, the lesions were maculopapular, with the formation of plaques similar to urticaria. *The photos were taken by the patient due to social isolation.

Curiously, the day after, the RT-PCR report was received, indicating a positive result for SARS-CoV-2. At that point, the authors concluded that the patient was mistakenly diagnosed with DF, and that this was a skin rash by SARS-CoV-2. After 20 days of absence from work and social activities, viral serologies showed SARS-CoV-2 IgM and IgG rapid tests reagents, and dengue IgM and IgG antibodies non-reagents (0.2 and 0.3, respectively) – confirming that this was not a viral co-infection, but a maculopapular rash of COVID-19.

The novel coronavirus started to spread in December 2019 in Wuhan, China. Several clinical manifestations have been associated with SARS-CoV-2 infection since then, including dermatological signs and symptoms. In a study with 171 patients, Freeman has described that the most common skin lesions morphologies have been morbilliform (22%), pernio-like (18%), urticarial (16%), and macular erythema (13%), which occurred after or concurrent with the systemic symptoms of COVID-19.^2^ Additionally, other articles have also documented enanthem, chickenpox‐like vesicles, and purpuric lesions as a clinical presentation of COVID-19.^3^

Misdiagnosis of other exanthematous diseases, such as DF, in patients with cutaneous manifestations of COVID-19, has also been reported. Joob and Wiwanitkit reported their experience in Thailand with a patient who was initially diagnosed with DF due to a rash with petechiae and flu-like symptoms, but after further investigation, SARS-CoV-2 infection was confirmed by RT-PCR.^4^

When this patient's investigation was initiated, in July 2020, Joinville (Santa Catarina, Brazil – 597,658 habitants) had approximately 5,000 confirmed cases of DF and 1,700 cases of COVID-19 in 2020. Thus, the authors emphasize that, in endemic regions for DF and COVID-19, the two diseases must be considered until proven otherwise. Unlike DF, which requires a vector, the coronavirus spreads very quickly through the air, and social isolation has been proven to block transmission. Finally, the authors warn of the possibility of co-infection of these two viruses in regions that are facing both epidemics at the same time; therefore, it is necessary to start early laboratory research for both diseases in order to guide the appropriate clinical management to the patient's condition.

## Financial support

None declared.

## Authors' contributions

Isabelle Pastor Bandeira: Approval of the final version of the manuscript; design and planning of the study; drafting and writing of the manuscript; critical review of the literature; critical review of the manuscript.

Beatriz Sordi Chara: Approval of the final version of the manuscript; design and planning of the study; drafting and writing of the manuscript; critical review of the literature; critical review of the manuscript.

Giovani Meneguzzi de Carvalho: Approval of the final version of the manuscript; design and planning of the study; drafting and writing of the manuscript; critical review of the literature; critical review of the manuscript.

Marcus Vinícius Magno Gonçalves: Approval of the final version of the manuscript; design and planning of the study; critical review of the literature; critical review of the manuscript.

## Conflicts of interest

None declared.
